# Granulocyte Colony-Stimulating Factor Alleviates Bacterial-Induced Neuronal Apoptotic Damage in the Neonatal Rat Brain through Epigenetic Histone Modification

**DOI:** 10.1155/2018/9797146

**Published:** 2018-02-01

**Authors:** Yung-Ning Yang, Yu-Tsun Su, Pei-Ling Wu, Chun-Hwa Yang, Yu-Chen S. H. Yang, Jau-Ling Suen, San-Nan Yang

**Affiliations:** ^1^Department of Pediatrics, E-DA Hospital, Kaohsiung, Taiwan; ^2^School of Medicine, I-Shou University, Kaohsiung, Taiwan; ^3^Graduate Institute of Medicine, College of Medicine, Kaohsiung Medical University, Kaohsiung, Taiwan; ^4^Joint Biobank, Office of Human Research, Taipei Medical University, Taipei, Taiwan; ^5^Research Center for Environmental Medicine, Kaohsiung Medical University, Kaohsiung, Taiwan; ^6^Department of Medical Research, Kaohsiung Medical University Hospital, Kaohsiung, Taiwan

## Abstract

Bacterial meningitis during the perinatal period may cause long-term neurological deficits. The study investigated whether bacterial lipopolysaccharide (LPS) derived from *E. coli.* led to neuronal apoptosis with an impaired performance of long-term cognitive function involving the activation of histone modification in the TNF-*α* gene promoter. Further, we looked into the therapeutic efficacy of granulocyte colony-stimulating factor (G-CSF) in a neonatal brain suffering from perinatal bacterial meningitis. We applied the following research techniques: neurobehavioral tasks, confocal laser microscopy, chromatin immunoprecipitation, and Western blotting. At postnatal day 10, the animals were subjected to LPS and/or G-CSF. The target brain tissues were then collected at P17. Some animals (P45) were studied using neurobehavioral tasks. The LPS-injected group revealed significantly increased expression of NF-*κ*B phosphorylation and trimethylated H3K4 in the *TNFA* gene promoter locus. Furthermore, the caspase-3, neuronal apoptosis expression, and an impaired performance in cognitive functions were also found in our study. Such deleterious outcomes described above were markedly alleviated by G-CSF therapy. This study suggests that selective therapeutic action sites of G-CSF through epigenetic regulation in the *TNFA* gene promoter locus may exert a potentially beneficial role for the neonatal brain suffering from perinatal bacterial-induced meningitis.

## 1. Introduction

Infectious disease especially meningitis during the neonatal or perinatal period may incur a high risk of causing brain damage and long-term development impairment such as cerebral palsy, schizophrenia, affective disorders, autism, and stroke [1]. Although many researchers had investigated how to prevent neurological sequelae after bacterial meningitis, its incidence does not seem to have significantly decreased over the last decade [2]. Current adjunctive therapeutic options are limited, and therefore ongoing research into the pathophysiology due to bacterial meningitis aims at providing the scientific basis for better adjunctive options [3].

Lipopolysaccharides, a well-known endotoxin and lipoglycans, are a membrane structural constituent of gram-negative bacteria. The immune response caused by lipopolysaccharide (LPS) due to bacterial infection may trigger the production of inflammatory cytokines. LPS stimulates the innate immune system to increase the expression of interleukin 1, NF-*κ*B, and tumor necrosis factor-alpha (TNF-*α*) [4, 5]. In the brain, LPS was found to bind to toll-like receptor 4 (TLR4), a kind of membrane protein, and then to trigger the nuclear factor-kappa B (NF-*κ*B) pathway that regulated the immune system [6] and controlled neuroprotection and neurodegeneration [7–9]. The activated NF-*κ*B pathway has been reported to induce the transcription of tumor necrosis factor-alpha (TNF-*α*) with LPS exposure [9, 10]. Furthermore, this induced TNF-*α* expression has been associated with acute or chronic inflammatory disease in the central nervous system (CNS) [11]. When it comes to bacterial infection, LPS exposure was reported to induce microglia to increase the secretion of TNF-*α* that increased neuron apoptosis via the caspase-3 pathway in the developing cerebral cortex [12]. In addition to increased apoptosis, LPS exposure was found to lead to decreased neurogenesis in the brain after [13, 14]. Therefore, bacterial meningitis may cause apoptosis of a neuron cell and decease neurogenesis though the NF-*κ*B and TNF-*α* pathways. The consequences of neonatal bacterial meningitis have been extensively investigated in the past, but the mechanisms related to long-term neurological dysfunctions have yet to be well documented.

Granulocyte colony-stimulating factor (G-CSF) is a kind of glycoprotein that has been used in patients with neutropenia in the past [15, 16]. Associated with the effects of neurogenesis, antiapoptosis, anti-inflammation, neurotrophy, and angiogenesis, G-CSF has neuroprotective effects on the mammalian adult brain [17–20]. G-CSF can stimulate bone marrow to produce stem cells and mobilize these stem cells from the bone marrow to the brain to induce neurogenesis in adult animals with stroke and improve functional outcomes [17, 18]. Additionally, G-CSF can reduce caspase-3 and thereby decrease apoptosis of cortical neurons [21]. Furthermore, G-CSF can also improve neurogenesis recovery and the decreased performance of long-term cognitive functions caused by perinatal hypoxia in the neonatal rat brain [22]. Moreover, the antiapoptotic effect of G-CSF in treating perinatal hypoxia pups has been observed via reduction in cleaved caspase-3 activity [23]. However, whether or not G-CSF alleviates bacterial-induced apoptosis in the neonatal rat brain still requires further investigation.

Epigenetic modification at the *TNFA* gene locus that modulates the expression of TNF-*α* mRNA and protein expression results from a concerted and complex network of regulation such as DNA methylation, histone modification, and chromatin remodeling [24, 25]. The studies frequently analyzed monocytes and macrophages. However, there is no published experimental evidence showing whether histone modification in the *TNFA* gene locus also regulates TNF-*α* mRNA and protein expression in the brain of pups with LPS. Hence, in this study, we also investigated whether LPS enhance TNF-*α* expression via this epigenetic modification and G-CSF alleviated LPS-mediated histone modifications at the *TNFA* gene locus. Furthermore, in order to examine the performance of long-term cognitive functions in pups with neonatal bacterial infection being treated with G-CSF, we also used an eight-arm radial maze task to investigate the pups' cued reference and spatial working memory.

## 2. Materials and Methods

### 2.1. Experimental Animal Protocols

The Animal Care and Use Committee at Kaohsiung Medical University and E-DA Hospital (Kaohsiung, Taiwan) approved all experimental procedures. Sprague-Dawley (SD) rats were provided with a 12 hr light/dark cycle and housed in the animal care facility. The animals were divided into four experimental groups as follows: vehicle-control (control group), LPS-treated-alone group (LPS group), G-CSF- (30 *μ*g/kg) alone group (G-CSF group), and LPS-treated plus G-CSF (30 *μ*g/kg) group (LPS + G group), on postnatal day ten (P10) that were considered compatible with human neonates [26]. To determine whether a single inflammatory event during development can influence long-term cognitive functions in later life, male rats were injected intracerebrally. The scalp was shaved, and a burr hole was drilled 1 mm caudal to the bregma and 2.0 mm lateral to the midline. LPS at the desired dosage was injected via a Hamilton microsyringe into the ventricle over a 5 min period. Control groups received isovolumetric i.c.v. injection of saline. Mice were maintained at 36°C with LPS (*Escherichia coli*, serotype O26:B6; 1 mg/kg) or pyrogen-free saline. Immediately after LPS injection, additional groups of rats were also injected with G-CSF (30 *μ*g/kg, single injection/day, subcutaneous injection) according to our previous study [22]. Brain tissue specimens of the target brain regions were collected on P17, and the memory functions and spatial learning were analyzed on P37–P58 employed as standard descriptions of adolescence [27].

### 2.2. Brain Tissue Slice Preparation

Bacterial meningitis may cause temporal lobe damage and epilepsy disorder [28, 29]. On P17, temporal lobe slices (400 *μ*m) were collected and immediately placed into artificial cerebrospinal fluid (ACSF) in a humidified atmosphere of 95% O_2_/5% CO_2_ at 34.0 ± 0.5°C in an incubation chamber for an equilibrium period of at least one hour [30]. To obtain the target brain regions, the brain slices were incubated with ice-cold oxygenated ACSF and then cut into tissue blocks (0.1 × 0.5 cm) within 15 seconds of slicing the tissue. The brain tissues were then immediately frozen at −80°C until analysis, and all tissue slices were only ever thawed once.

### 2.3. Immunoblotting

After our customized treatments, the cells were lysed into cell lysis buffer (10 mM Tris (pH 7.4), 150 mM NaCl, 2 mM EDTA, 0.2% Triton X-100, 1 mM PMSF, and 1× protease inhibitor mixture). The protein concentration was then determined by using the BCA assay (Thermo Scientific, Rockford, IL, USA). Cell lysates were separated on SDS-PAGE and then transferred to nitrocellulose membranes. They were probed with an antibody against NF-*κ*B (AbFrontier/LF-PA0062), pNF-*κ*B (AbFrontier/LF-PA20342), TNF-*α* (SANTA CRUZ/SC-1351), and caspase-3 (Cell Signaling #9662). Our secondary antibodies were either rabbit anti-mouse IgG or goat anti-rabbit IgG (1 : 3000), depending on the primary antibody. Finally, the immunoreactive proteins were detected using the BioSpectrum 810 Imaging System (UVP).

### 2.4. Chromatin Immunoprecipitation Assay (ChIP)

As in our previous study [31], 5 × 10^5^ cells were treated for 10 min at room temperature with 1% formaldehyde, followed by sonication of DNAs and the immunoprecipitation of chromatin and then purification with the ChIP kit (ChIP kit number 17-295; Upstate Biotechnology, Lake Placid, NY, USA). Probes and primers were designed by analyzing the proximal promoter and intronic enhancer regions of the TNFA gene for the polymerase chain reaction (PCR) amplification of ChIP products. These regions included the following subregions relative to the transcription start site: TNF1 (T1; −2667 to −2686), TNF2 (T2; −2183 to −2202), TNF3 (T3; −1653 to −1672), TNF4 (T4; −174 to −496), and TNF5 (T5; −205 to −224). PCRs were performed on the ABI 7700 TaqMan thermocycler (Applied Biosystems).

### 2.5. Evaluation of Apoptosis

We performed double immunofluorescence analysis using laser-scanning confocal microscopy to analyze apoptosis in neuron cells in tissue. The staining protocol was described in a previous study [22] and briefly summarized in the following sentences. The brain samples were coronally cut at 30 *μ*m and stained with antineuronal nuclei (NeuN) (clone A60, MAB377; Chemicon, Temecula, CA, USA) for neuron cell identification and with TUNEL (Sigma-Aldrich, 11684795910) for DNA break detection. TUNEL was performed according to the manufacturer's instruction. Secondary amplification was performed using a Cy3-conjugated anti-mouse antibody for NeuN staining and fluorescein-conjugated antibody for TUNEL staining. Negative controls for NeuN and TUNEL double labeling were obtained by omitting either the anti-NeuN antibody or the TdT enzyme in the TUNEL reaction. Images of epifluorescence were collected on a Leica (Nussloch, Germany) DMIRE2 microscope, and images of confocal fluorescence were collected using a Zeiss (Thornwood, NY, USA) LSM510 microscope.

### 2.6. Eight-Arm Radial Maze Task

Spatial memory tasks such as eight-arm radial maze and Morris water maze are tools used in evaluating temporal lobe lesions [32]. As in our previous study [33], an eight-arm radial maze was used to train rats on two memory tasks. In this test, two memory deficits can be recorded: (1) working memory error (WME): a rat goes to an arm with bait that it has already visited, and (2) reference memory error (RME): a rat visits an arm with no bait. If the forepaws of the rat crossed the midline of the arm, this was defined as having visited that arm. The rats were defined as having successfully completed the cued reference memory and spatial working memory tasks when there were no RMEs or WMEs for at least 2 days within a 3-day period.

### 2.7. Statistical Analysis

All data are presented as mean ± SEM. Statistical differences were determined by using one-way ANOVA followed by Bonferroni's *t*-test for post hoc multiple comparisons. A statistical significance level of *p* < 0.05 was applied to all tests.

## 3. Results

### 3.1. G-CSF Alleviated the Enhancement of Phosphorylated NF-*κ*B p65 in the Brain of Pups with LPS Treatment

**Figure 1:**
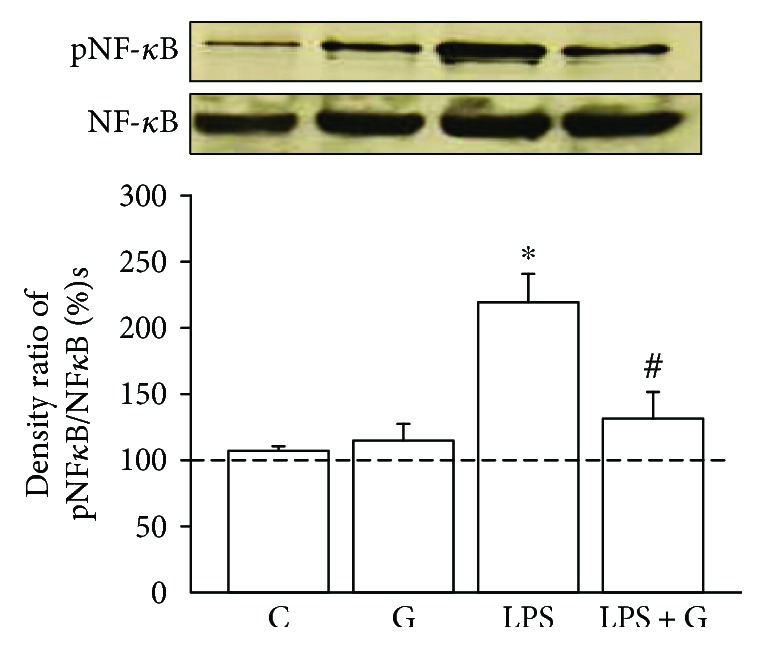
G-CSF alleviated the elevated pNF-*κ*B due to LPS treatment. Western blot analysis was used to detect the phosphorylated NF-*κ*B (pNF-*κ*B) p65 levels in the brain cell. The experimental groups are indicated as follows: vehicle-control (C), LPS group (LPS), G-CSF group (G), and LPS + G group (LPS + G) (*n* = 5 animals for each experimental group). The results were expressed as relative level of pNF-*κ*B to total NF-*κ*B. In the LPS group, the ratio of pNF-*κ*B to total NF-*κ*B was significantly higher compared to that in the LPS + G group and control group. ^∗^*p* < 0.05 compared with the control group. ^#^*p* < 0.05 compared with the LPS group. Data are mean ± standard error of mean.

Pups treated with LPS increased the expression of phosphorylated NF-*κ*B p65 in the brain. There was a significant enhancement of phosphorylated NF-*κ*B p65 (Figure 1) in the LPS group compared with the control group (LPS 219.4 ± 21.1% versus control 107 ± 3.2%, *p* < 0.05). There was also no significant difference in the LPS + G group compared to the control group. The results suggest that the changes of the rat brain experimentally exposed to LPS involved the NF-*κ*B signaling pathway and that increased phosphorylated NF-*κ*B expression could be alleviated by G-CSF therapy.

### 3.2. G-CSF Alleviated the Enhancement of TNF-*α* in the Brain of Neonatal Rats Treated with LPS

**Figure 2:**
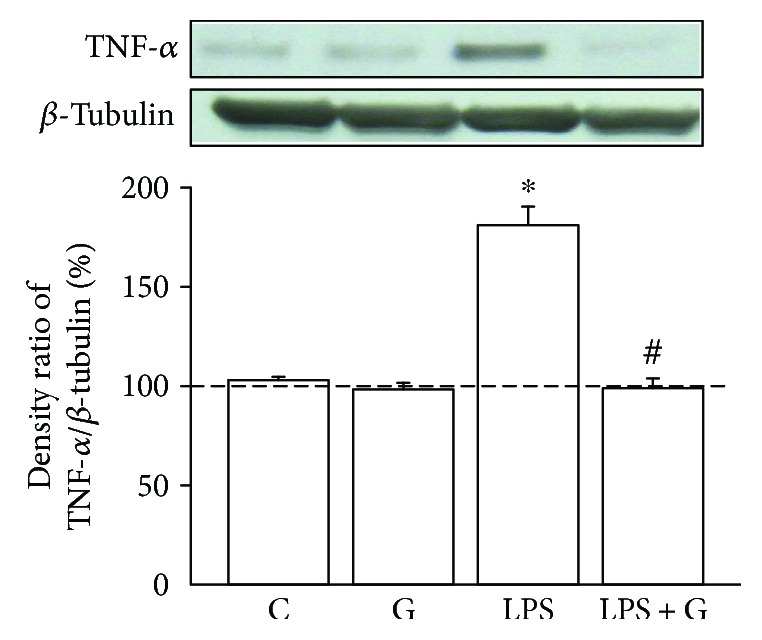
G-CSF alleviated the elevation of TNF-*α* expression levels. Effect of G-CSF on LPS-induced production of TNF-*α*. The experimental groups are indicated as follows: vehicle-control (C), LPS (LPS), G-CSF (G), and LPS + G (LPS + G) (*n* = 5 animals for each experimental group). The level of TNF-*α* expression was elevated in the LPS group compared with the control group. There were no significant differences between the LPS + G-CSF and control groups. ^∗^*p* < 0.05 compared with the vehicle-control group. ^#^*p* < 0.05 compared with the LPS group. *β*-Actin levels performed in parallel served as controls.

To demonstrate that LPS influenced the downstream signaling pathway of NF-*κ*B, we examined the concentrations of TNF-*α*. The LPS group showed a significantly increased expression of TNF-*α* (Figure 2) compared with the control group (LPS 181.5± 9.2% versus control 103 ± 1.6%, *p* < 0.05). In the LPS + G group, TNF-*α* expression was significantly reduced compared with that in the LPS group. These findings reveal that G-CSF might be effective for alleviating the increase in TNF-*α* expression in the brain of pups experimentally exposed to LPS.

### 3.3. LPS-Mediated Histone Modifications at the TNFA Gene Locus

**Figure 3:**
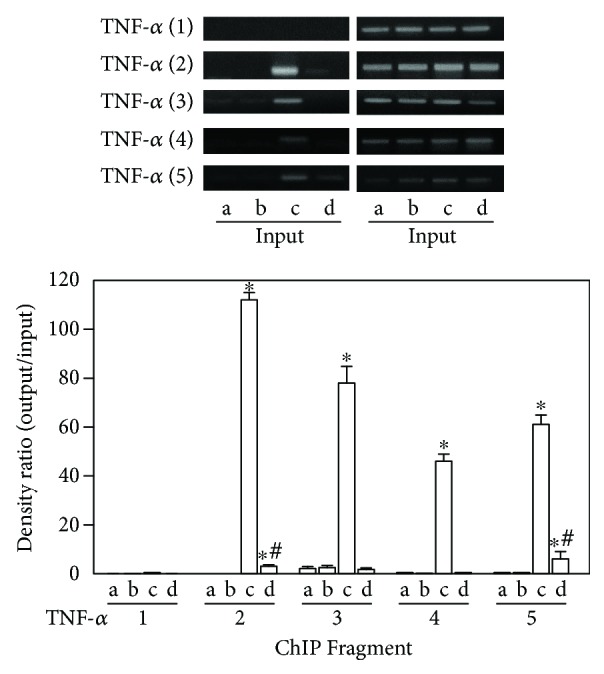
G-CSF therapy involved histone acetylation in the regulatory effect of LPS treatment on TNF-*α* expression. ChIP analyses of the relative levels of acetylated H3 at the *TNFA* gene locus included the following subregions relative to the transcription start site: TNF1 (T1; −2667 to −2686), TNF2 (T2; −2183 to −2202), TNF3 (T3; −1653 to −1672), TNF4 (T4; −174 to −496), and TNF5 (T5; −205 to −224). The groups (a–d) are as follows: (a) control, (b) G-CSF, (c) LPS, and (d) LPS + GCSF. The relative levels were normalized to the input DNAs and are shown as mean ± SD of the study subjects. The level of histone acetylation was significantly higher in the LPS group compared with the control group. ^∗^*p* < 0.05 compared with the control group. ^#^*p* < 0.05 compared with the LPS group.

Epigenetic modifications regulate the expression of TNF-*α* in response to acute stimulations such as by LPS [24, 34]. A recent study analyzing macrophages and monocytes demonstrated that acetylated H3 and H4 mark active transcription in the *TNFA* gene locus [24]. To examine whether the *TNFA* gene locus underwent histone modifications in neurons as the result of the LPS treatment and NF-*κ*B activation, we performed ChIP analyses of the pups' brains treated with LPS, using PCR primers corresponding to five subregions (TNF1–5) in the TNFA promoter of the *TNFA* gene. The results showed that compared to the vehicle-control group, the neonatal rat brains treated with LPS significantly presented histone modifications at the *TNFA* gene locus. In Figure 2, upregulated TNF-*α* expression was noted in LPS-treated neurons. ChIP analyses revealed increased levels of trimethylated H3K4 at the promoter subregions, TNF2–5, of the *TNFA* gene in the neonatal rat brain of the LPS group (Figure 3). Further, a significant increase in trimethylated H3K4 at the TNF2–5 promoter subregions (Figure 3) was seen in the LPS + GSF group. This indicates that G-CSF can alleviate the brain damage of perinatal LPS exposure via epigenetic regulation in the *TNFA* gene promoter locus.

### 3.4. G-CSF Decreased Caspase-3 Activity Enhanced by LPS Treatment in the Brains of Neonatal Rats

**Figure 4:**
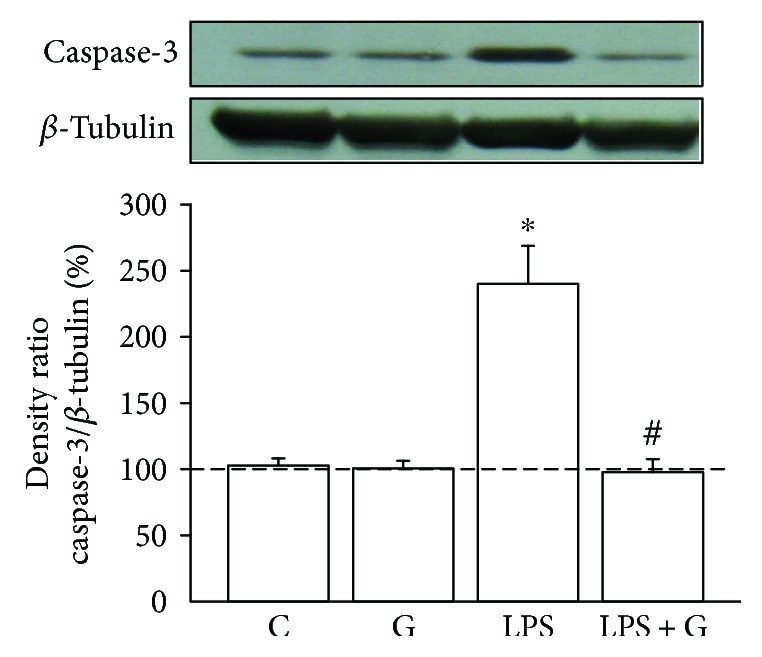
G-CSF alleviated the elevation of caspase-3 activity in pups treated with LPS. Effect of G-CSF on caspase-3 activity in the brain of pups experimentally exposed to LPS. The experimental groups are indicated as follows: vehicle-control (C), LPS (LPS), G-CSF (G), and LPS + G (LPS + G) (*n* = 5 animals for each experimental group). Caspase-3 activity was significantly elevated in the LPS group compared with the vehicle-control group. The caspse-3 activity also showed no significant differences between the LPS + G and control groups. ^∗^*p* < 0.05 compared with the control group. ^#^*p* < 0.05 compared with the LPS group. *β*-Actin levels performed in parallel served as controls.

Since we observed that G-CSF had anti-inflammatory effects and was regulated via epigenetic modification, we then investigated the antiapoptotic effect of G-CSF. As can be seen in Figure 4, caspase-3 activity in the LPS group was significantly higher than that in the control group (LPS 240.6 ± 28.1% versus control 103.4 ± 5.1%, *p* < 0.05). In the LPS + G group, caspase-3 activity was significantly lower compared with that in the LPS group, but no statistical significance was reached with respect to the control group. Therefore, G-CSF was found to have an antiapoptotic effect by decreasing the intensified caspase-3 activity in pups experimentally treated with LPS.

### 3.5. Evaluation of the Antiapoptotic Effect of G-CSF in Pup's Brain after Treatment with LPS

**Figure 5:**
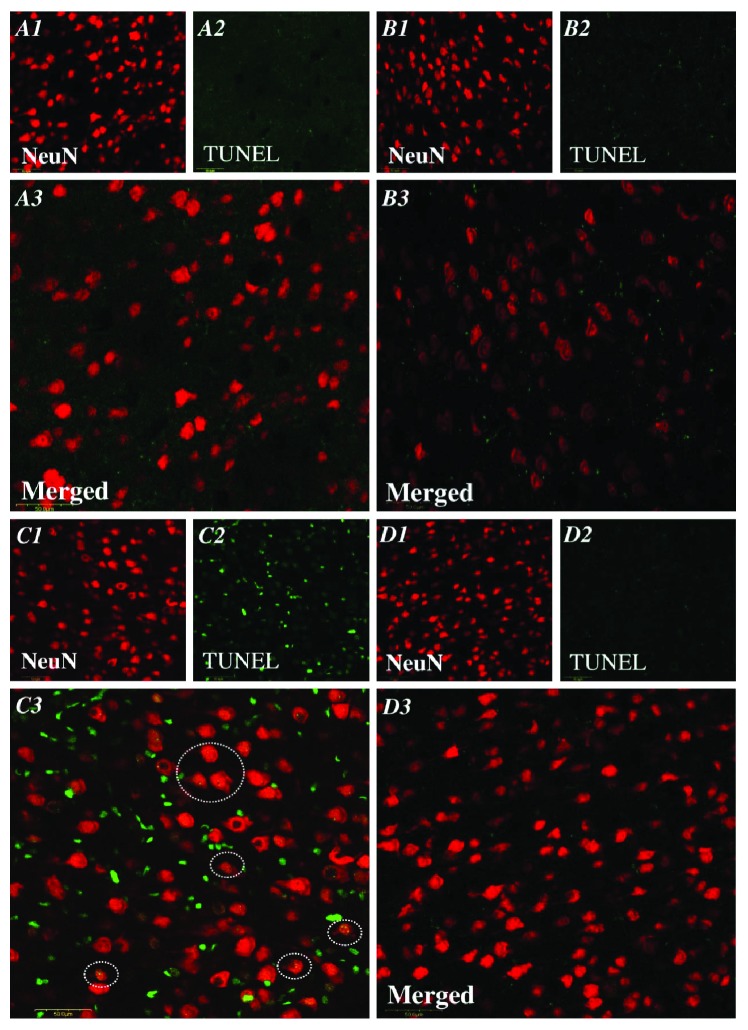
Effects of G-CSF on the increasing expression of neuronal apoptosis with LPS treatment. Colocalization of neuronal nucleus- (NeuN, red for neuron identification) and TUNEL- (green for apoptotic cells) positive cells, as assessed on P17, was identified by double immunofluorescence staining under laser-scanning confocal microscopy. The NeuN-positive cells (1), TUNEL-positive cells (2), and the combined pictures of the NeuN- and TUNEL-positive cells (3) are as follows: vehicle-control (A1–3), G-CSF (B1–3), LPS (C1–3), and LPS + G (D1–3) (30 mg/kg). The dotted circles reveal the representative neuronal apoptosis. Bar = 50 *μ*m.

**Figure 6:**
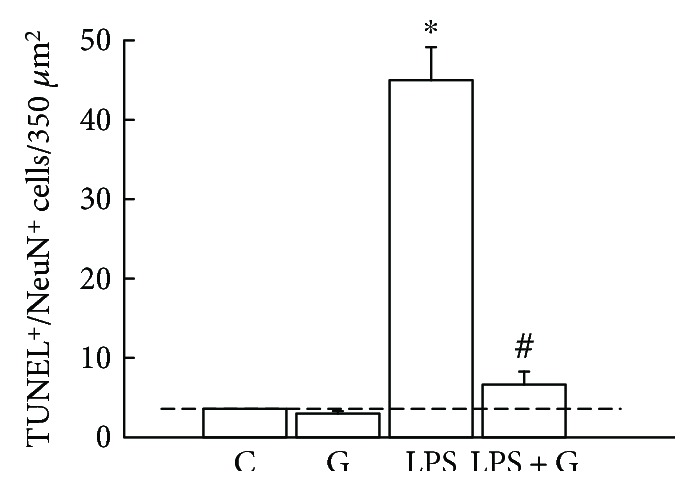
Quantified analysis of the effects of G-CSF on the increased expressions of neuronal apoptosis with LPS treatment. The experimental groups are indicated as follows: control (C), G-CSF (G), LPS (LPS), and LPS + G (LPS + G) (animals for each experimental group). Colocalization of neuronal nucleus- (NeuN-) positive and TUNEL-positive cells indicates apoptotic neuronal cells. ^∗^*p* < 0.05 compared with the vehicle-control group. ^#^*p* < 0.05 compared with the LPS group. Data are presented as mean ± standard error of the mean.

To confirm the decrease in caspase-3 activity, a confocal microscope was used to determine whether the G-CSF therapy alleviated the apoptosis in the brain of pups after LPS. We used neuronal marker NeuN (for mature neurons) and TUNEL to assess the level of apoptosis in the brain of pups. A declining trend in the coexpression of NeuN-positive cells with TUNEL-positive cells was revealed in the neonatal LPS group (Figure 5(c)) compared with the control rats (Figure 5(a)). In contrast, the increase in the number of TUNEL-positive cells colocalizing with NeuN-positive cells was observed in the LPS + G group (Figure 5(d)). In a quantitative analysis (Figure 6), the average number of cells exhibiting double staining with NeuN-positive and TUNEL-positive cells within the counted areas was significantly higher in the LPS group compared to the control group (LPS 45.1 ± 4.2 cells/*μ*m^2^ versus control 3.6 ± 0.1 cells/*μ*m^2^, 5 slides per animal, 5 animals, *p* < 0.05). In the LPS + G therapy, there was no significant difference with respect to the control group, but there was a significant decrease in the effect of apoptosis with respect to the LPS group. These results revealed that G-CSF therapy had a significant efficacy against the apoptosis caused by neonatal LPS treatment.

### 3.6. Using Eight-Arm Radial Maze Task to Evaluate Long-Term Cognitive Function

**Figure 7:**
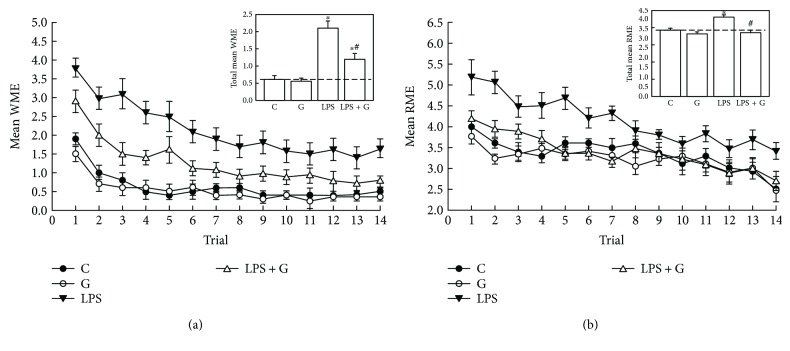
Impairment of the long-term performance of working and cued reference memory was found using an eight-arm radial maze task after LPS exposure. (a) One trial/day over 15 trials and average amount of working memory errors. The inset box reveals the average total mean working memory errors. (b) One trial/day over 15 trials and average amount of cued reference memory errors. The inset box reveals the average of total mean reference memory errors. Control group (*n* = 10 animals), G-CSF group (*n* = 10 animals), LPS group (*n* = 10 animals), and LPS + G group (*n* = 10 animals) are labeled as C, LPS, G, and LPS + G, respectively. Statistical analysis of the long-term performance of the working and cued reference memory revealed that the rats in the LPS group significantly differed from those in the vehicle-control and LPS + G groups. ^∗^*p* < 0.05 compared with the control group. ^#^*p* < 0.05 compared with the LPS group

Figure 7 shows the long-term cognitive deficits in the working and cued reference memory tasks in the rats postneonatally treated with LPS after repeated daily training with the eight-arm maze task. Compared with the control group, the LPS group had more mean WMEs (*n* = 10 animals, *p* < 0.05, Figure 7(a)) and RMEs (*n* = 10 animals, *p* < 0.05, Figure 7(b)) in the first few days of life. In the LPS + G group, G-CSF therapy alleviated the decrease in mean RME and WME compared with that in the LPS-treated-alone group. Furthermore, the LPS group exhibited a significant increase in total mean RMEs and total mean WMEs compared with both the control and LPS + G groups. These results suggest that LPS exposure of the neonatal brain can lead to a decline in the ability of spatial memory formation and the insult can be alleviated after G-CSF therapy.

## 4. Discussion

In this study, our results show that the G-CSF therapy was useful in treating bacterial meningitis of neonates. Firstly, G-CSF alleviated the LPS-induced brain damage through the NF-*κ*B pathway. Secondly, the effect of G-CSF therapy was via epigenetic regulation in the *TNFA* gene promoter locus by selective therapeutic action sites. Thirdly, G-CSF also diminished the increase in caspase-3 expression and apoptosis. Fourthly, working and cued reference memory impairments due to LPS exposure were alleviated by G-CSF therapy.

LPS, associated with gram-negative bacteria, was recognized by TLR4 to induce brain injury and increase the TNF-*α* expression via the NF-*κ*B pathway [9]. To activate the NF-*κ*B pathway, it depended on I*κ*B kinase phosphorylation of the inhibitory molecules and phosphorylation of the P65 protein. Then, the phosphorylation of the P65 protein was translocated into the nucleus [35, 36]. This phosphorylated P65 protein has the ability to recruit histone acetyltransferases and lead to the production of proinflammatory factors, such as TNF-*α*. This study's findings are compatible with those of a previous study in that the LPS-exposed neonatal brain enhanced the expression of phosphorylated NF-*κ*B p65 and TNF-*α* [35]. Several studies have established the possible mechanism explaining how LPS affects the production of TNF-*α* in adults. In this study, we provided evidence of this pathway in the neonatal rat brain.

Trimethylation of H3K4 played an important role in the initial recruitment of a methyltransferase complex (which includes a methyltransferase, mixed lineage leukemia (MLL), and WD repeat domain 5 (WDR5) protein scaffolds), which in turn mediates dimethylation to trimethylation conversion of histone H3 at K4 [37]. MLL1, one of the six members of the MLL family, has been reportedly associated with the P65 protein of the NF-*κ*B family [38]. In the innate immune system, a previous study reported that the NF-*κ*B regulated its downstream gene transcription (TNF-*α*) via MLL1 [38]. In the CNS, LPS-regulated TNF-*α* expression might also occur via NF-*κ*B and its downstream epigenetic modulation. Although there are very few data about how LPS regulate TNF-*α* in the brain, LPS-regulated TNF-*α* via NF-*κ*B in brain microglia has been shown in some brain disorders [39]. This study's findings can shed some further light on this issue. We showed that LPS affected TNF-*α* expression via NF-*κ*B and its downstream reaction of variable patterns of histone modifications in neuron cells. Additionally, the neuroprotective effect of G-CSF not only alleviated the increase in NF-*κ*B expression but also elevated the histone trimethylation of H3K4. Therefore, at last in part, the LPS and G-CSF both regulated the TNF-*α* expression at the epigenetic level. Furthermore, the significant enhancement of trimethylated H3K4 was found at the TNF2–5 promoter subregions but not at all the TNF promoter subregions after LPS exposure. Moreover, the G-CSF therapy cannot repair all the damages caused by LPS treatment.

Caspases are a group of cysteinyl-aspartate-specific proteases that have a marked influence on apoptotic cell death [40, 41]. Pups treated with LPS may induce proinflammatory cytokine release, such as TNF-*α*. The increased expression of TNF-*α* protein may result in the increase in caspase-3 cleavage and cause neuron apoptosis [42]. Also, caspase-3 is considered to be the effector caspase that is activated by caspase-8, caspase-9, and caspase-10. Since G-CSF has been reported to modulate LPS-induced apoptosis in microvascular endothelial cells via caspase-3 [43], we investigated caspase-3 activity to explore the protective effect of G-CSF in LPS-induced brain injury. Even though there have been many recent studies investigating the way to decrease the damage of neonatal meningitis, their results have been limited. Our results demonstrate that G-CSF decreased caspase-3 activation and provided neuroprotective effects on fetal rat brain following experimental neonatal inflammation.

G-CSF has neuroprotective effects through the pathway of anti-inflammation, antiapoptosis, and neurogenesis in the CNS [44, 45]. Brain inflammation may cause blood-brain barrier injury and lead to leukocyte migration into injured tissue [44]. The anti-inflammatory effects of G-CSF happened though the pathway to reduce the level of cytokines, such as TNF-*α* and interlukin-1*β*, secreted by leukocytes, and to reduce T cell migration to the CNS [46]. G-CSF has also been noted to activate Bcl-2 and bcl-xl protein which reduces caspase-3 to prevent apoptosis through the JAK2/STAT3 pathway [21, 46]. This study provided evidence in a rat model that G-CSF can reduce the inflammatory response by reducing TNF-*α* via epigenetic regulation and can also have an antiapoptotic effect by reducing caspase-3 in neonatal meningitis.

In the CNS, neural stem cells, capable of self-renewal, are able to differentiate into neurons, oligodendrocytes, and astrocytes. When the brain suffers an injury or inflammatory accident, the permeability of the blood-brain barrier may change and then allow the G-CSF-induced neutrophil to pass [46]. This has been shown to stimulate the bone marrow to produce stem cells and induce their mobilization from the bone marrow to the brain, which will then induce neurogenesis in adult animals with ischemic or nonischemic disorder to improve functional outcomes [17, 18, 21, 23, 47–50]. Initially, G-CSF mobilizes hematopoietic progenitor cells from the bone marrow to peripheral blood, which then migrate to the site of neuronal damage through the CXC chemokine receptor 4 (CXCR4)/stromal cell-derived factor-1 (SDF-1) system [51]. Via the PIK/Akt pathway, G-CSF then enhances neurogenesis and neuroblast migration after stroke [46]. In addition, phosphorylated CREB^Ser-133^ (*p*CREB^Ser-133^), a DNA-binding transcription factor, plays an important role in the regulation of some immediate-early genes [52]. In an earlier study, we reported that G-CSF therapy alleviates the decrease in pCREB^Ser-133^ and PSD-95 with NMDAR subunits within the CA1 region of the hippocampus after perinatal hypoxia and that G-CSF therapy provides beneficial effects for long-term cognitive deficits [53]. Given that LPS has been reported to modulate the NMDA receptor of glial cells with TNF-*α* [54], G-CSF might also work in treating brain damage from LPS exposure.

In recent years, our group has also focused on the neurogenesis effect of G-CSF on pups to investigate the therapy of perinatal hypoxia-ischemia brain injury [22]. G-CSF not only improved neurogenesis but also improved long-term cognitive function in the pups with experimentally induced perinatal hypoxia. However, whether the neuroprotective effects of G-CSF are also displayed in multiple brain regions after experimental LPS exposure remained unknown until the present study. Here, we have shown the efficacy of G-CSF therapy in the neonatal rat brain experimentally treated with LPS.

## 5. Conclusion

This study offers a new insight into how G-CSF therapy is able to recover the damage caused by LPS exposure through the decline of inflammation and apoptotic reaction and how G-CSF therapy can, at least in part, improve long-term functional outcomes (in terms of the improvement of impaired performance in spatial working and cued reference memory). And even more importantly, the effect of G-CSF was shown to occur through epigenetic regulation of the TNFA gene. In addition, environment enrichment may provide a better outcome in LPS-exposed subjects, and early rehabilitation might be useful. This study suggests a new potential neuroprotective strategy that may be well tolerated with fewer side effects on the neonatal brain with bacterial meningitis. Although we are far from being able to apply this therapy in human neonates, our results provide an insight into neurological function defects in patients after neonatal bacterial infection.
